# Evolution of Pentameric Ligand-Gated Ion Channels: Pro-Loop Receptors

**DOI:** 10.1371/journal.pone.0151934

**Published:** 2016-03-17

**Authors:** Mariama Jaiteh, Antoine Taly, Jérôme Hénin

**Affiliations:** Laboratoire de Biochimie Théorique, Institut de Biologie Physico-Chimique, CNRS and Université Paris Diderot, Paris, France; Laboratoire Oceanologique de Banyuls sur Mer, FRANCE

## Abstract

Pentameric ligand-gated ion channels (pLGICs) are ubiquitous neurotransmitter receptors in Bilateria, with a small number of known prokaryotic homologues. Here we describe a new inventory and phylogenetic analysis of pLGIC genes across all kingdoms of life. Our main finding is a set of pLGIC genes in unicellular eukaryotes, some of which are metazoan-like Cys-loop receptors, and others devoid of Cys-loop cysteines, like their prokaryotic relatives. A number of such “Cys-less” receptors also appears in invertebrate metazoans. Together, those findings draw a new distribution of pLGICs in eukaryotes. A broader distribution of prokaryotic channels also emerges, including a major new archaeal taxon, Thaumarchaeota. More generally, pLGICs now appear nearly ubiquitous in major taxonomic groups except multicellular plants and fungi. However, pLGICs are sparsely present in unicellular taxa, suggesting a high rate of gene loss and a non-essential character, contrasting with their essential role as synaptic receptors of the bilaterian nervous system. Multiple alignments of these highly divergent sequences reveal a small number of conserved residues clustered at the interface between the extracellular and transmembrane domains. Only the “Cys-loop” proline is absolutely conserved, suggesting the more fitting name “Pro loop” for that motif, and “Pro-loop receptors” for the superfamily. The infered molecular phylogeny shows a Cys-loop and a Cys-less clade in eukaryotes, both containing metazoans and unicellular members. This suggests new hypotheses on the evolutionary history of the superfamily, such as a possible origin of the Cys-loop cysteines in an ancient unicellular eukaryote. Deeper phylogenetic relationships remain uncertain, particularly around the split between bacteria, archaea, and eukaryotes.

## Introduction

Pentameric ligand-gated ion channels (pLGICs) mediate fast synaptic transmission in the nervous system of animals with bilateral symmetry (Bilateria), where they are ubiquitous and known as Cys-loop receptors [1–4]. Each receptor is a fivefold symmetric or pseudosymmetric transmembrane assembly of protein subunits surrounding a central pore that is selective of either cations or anions.

The idea that pLGICs are ancient enough to predate eukaryotes, and that they may have prokaryotic relatives was formulated in 1990 by Cockcroft et al. [5]. No pLGIC was known outside metazoan Cys-loop receptors until the discovery of their prokaryotic homologues by Tasneem et al. [6]. Since prokaryotic receptors lack the eponymous cysteine residues, the Cys-loop family was then superseded by the superfamily that became known as pLGICs. Two prokaryotic pLGICs have been cloned and characterized functionally [7, 8] and were the choice models for high-resolution structural studies [9–13] that paved the way for the recent successes with animal Cys-loop receptors [14–16].

The currently documented taxonomic distribution of pLGICs is that established by Tasneem et al. [6], that is, Cys-loop receptors are ubiquitous in Bilateria and other pLGICs are found sporadically in several bacterial taxons and one archaeal genus. Subsequent work by the same group [17] refined the methods but did not fundamentally alter that picture, while Corringer et al. [18] reported that cursory searches of genome databases revealed more bacterial genes likely to belong to the superfamily. Tasneem et al. interpreted the taxonomic distribution as indicating a complex evolutionary history involving multiple lateral transfers and frequent gene loss. The case of non-bilaterian metazoans has received relatively little attention, presumably due to the scarcity of genome data, although pLGICs have been documented in the cnidarians *Hydra*[19] and *Nematostella*[20, 21]. While the discovery of prokaryotic homologues has provided a fascinating glimpse on the evolutionary origin of animal pLGICs, many questions remain open, among which the ancestry of animal Cys-loop receptors, the reasons for the sparse yet broad distribution of pLGICs in prokaryotes, and the biological roles of those microbial proteins, none of which has been studied *in vivo*.

Here we exploit the ever-growing body of genomic data to expand our knowledge of pLGICs throughout the tree of life, focusing on taxonomic groups where they are less well characterized. We extend the inventory of members of the superfamily across all kingdoms of life by performing remote homology searches in protein sequence databases. As metazoan members are better known, we focus on unicellular organisms, and find previously unreported pLGICs in unicellular eukaryotes (called “protists” below for brevity), as well as a broader distribution of prokaryotic channels including a major new archaeal taxon. We also detect and investigate metazoan pLGICs lacking the eponymous Cys-loop cysteines. We construct a multiple sequence alignment of a broadly distributed set of pLGIC sequences, and use it to derive a maximum-likelihood phylogenetic tree that suggests new hypotheses on the evolutionary history of this ancient superfamily. Finally, we discuss the questions that remain unsolved, particularly in light of the difficulty of inferring ancient evolutionary relations based on limited phylogenetic signal.

## Results

### Taxonomic distribution

**Fig 1:**
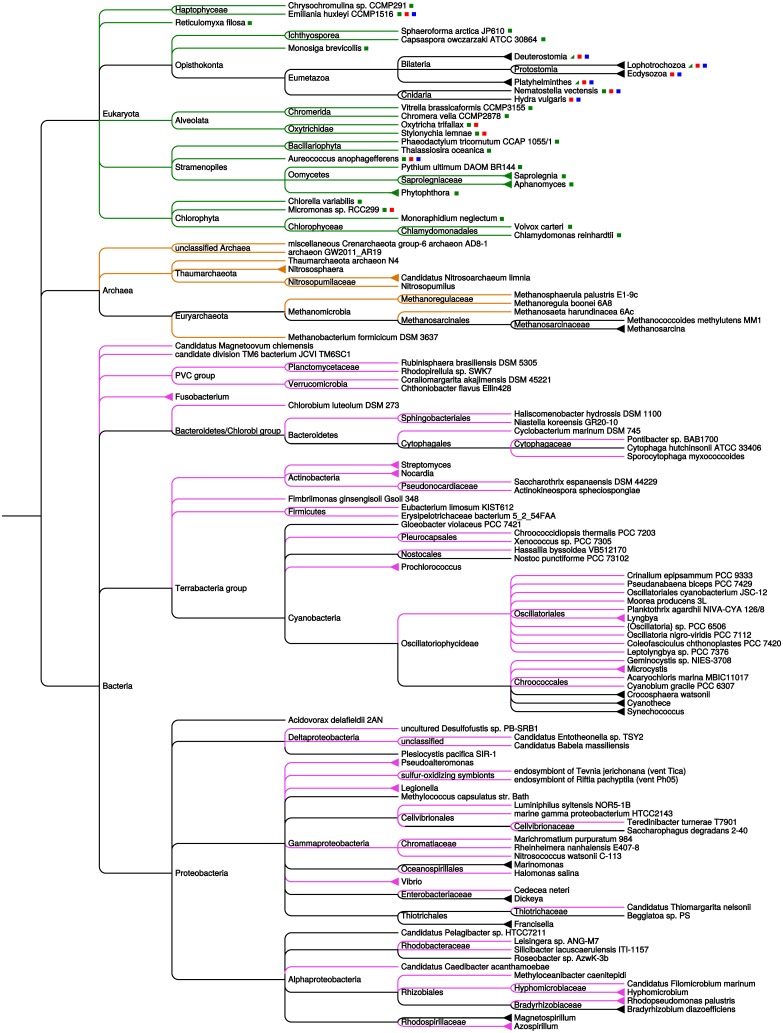
Phylogenetic tree of organisms whose genomes contain predicted Pro-loop receptors. Colored branches represent taxons that were not discussed previously in the pLGIC literature (green: unicellular eukaryotes, orange: archaea, magenta: bacteria). Colored squares next to eukaryotic taxa indicate the types of pLGICs present (green: Cys-less, blue: cationic-type Cys-loop, red: anionic-type Cys-loop); the half green squares next to metazoan taxons indicate the presence of Cys-less pLGICs in some species. The tree is extracted from NCBI Taxonomy.

We find predicted pLGICs in new prokaryotic organisms including several Archaea, and perhaps more surprisingly, in a number of unicellular eukaryotes. Compared with the work of Tasneem et al. [6], we find pLGICs in 61 new bacterial genera, 10 new archaeal genera, and 22 protist genera. This new taxonomic distribution of pLGICs is Illustrated in Fig 1, wherein each pLGIC-possessing taxon is placed within a tree of life and colored branches indicate new taxons.

The main novel finding is the existence of pLGIC genes in a wide array of protists. Those include Stramenopiles (a large group including marine organisms such as diatoms, and Oomycetes), Chlorophyta (green algae such as *Chlamydomonas* and *Chlorella*), Opisthokonta (the group that also includes animals and Fungi), and Alveolata. Noteworthily, we detect no pLGICs in multicellular plants (relatives of Chlorophyta) or Fungi (Opisthokonta). Thus, metazoans remain the only multicellular organisms known to possess pLGICs. Within Opisthokonta, pLGIC genes are found in the Choanoflagellate *Monosiga brevicollis*, one of the closest known relatives of metazoans, [22] and *Capsaspora owczarzaki*, in the slightly more distant taxon Filasterea [23]. In contrast, the one other complete genome from a Choanoflagellate, *Salpingoeca rosetta*[24], yields no hit.

The new bacterial pLGIC genes belong to species in many taxons, and diverse ecological niches: marine, soil, plant pathogens, and a few human pathogens. Bacterial species associated with humans include *Erysipelotrichaceae bacterium*, which was isolated from the gut of patients with Crohn’s disease; human pathogens *Francisella tularensis*, *Fusobacterium varium*, and *Legionella drancourtii*.

Many new archaeal genomes have been sequenced since the work of Tasneem et al. Whereas the two pLGICs of *Methanosarcina* pinpointed by that work were taxonomically isolated and offered little basis for evolutionary interpretation, this larger set supports a more substantial analysis of molecular phylogeny. We find pLGIC genes in new archaeal species in Euryarchaeota and in a number of Thaumarchaeota [25]. Taxonomic coverage of those groups is very difficult to measure, as most hits come from datasets that are not annotated as whole genomes. Notably, we find no hit in the phylum Crenarchaeota, despite the presence of dozens of genomes (complete or partial) in databases. Finally, as this work was being completed, the taxon Lokiarchaeota was described based on metagenomic data, and presented as a link between archaea and eukaryotes [26]. Extending our search to the set of predicted proteins for the candidate species *Archaeon loki* did not yield significant hits.

Our representative set of metazoan genomes includes two non-bilaterian species: *Hydra vulgaris* and *Nematostella vectensis* (both Cnidaria). Both have genes in the anionic and cationic groups of Cys-loop receptors. *Hydra* has two Cys-loop receptors, automatically annotated as *γ*-aminobutyric acid (GABA_*A*_, Uniprot ID T2MFN2) and nicotinic acetylcholine receptor (nAChR, T2MCD8). A taxonomically unrestricted search in UniprotKB yields no other examples of non-bilaterian pLGICs. In particular, there is no hit in the genomes of basal metazoans Placozoa [27], Porifera [28], and Ctenophora [29]. Of these three phyla, only Ctenophora possess a nervous system, which has been proposed to have evolved independently from those of Bilateria and Cnidaria, which share a common origin [29].

pLGICs of both *Hydra*[19] and *Nematostella*[20] have been discussed before. The authors who reported the *Hydra* genome found the expression pattern of the nAChR gene to be compatible with a function in neuromuscular signaling [19]. They find the neuromuscular junction of *Hydra* to possess many, but not all, of the molecular components found in the equivalent bilaterian system. Neither the GABA_*A*_-like receptor of *Hydra*, nor the pLGICs of *Nematostella* have been characterized experimentally.

### Multiple sequence alignment

The complete multiple sequence alignment is provided as S1 Dataset. It contains 561 protein sequences: 218 metazoan (among which 69 Cys-less), 193 eubacterial, 24 archaeal and 126 from protists. The alignment has 3405 positions, discarding segments at the N and C-termini that do not align with the pLGIC domains. The variable M3M4 cytoplasmic region accounts for 1170 gap-rich positions. The “core” of the alignment includes about 320 well-aligned positions (a typical pLGIC sequence length without the cytoplasmic loop), interspersed with about 1915 poorly aligned and gap-rich positions. Despite the high noise level, significant structural elements emerge as less gapped blocks, most notably the four transmembrane helices and some beta-strands of the extracellular domain (ECD).

**Fig 2:**
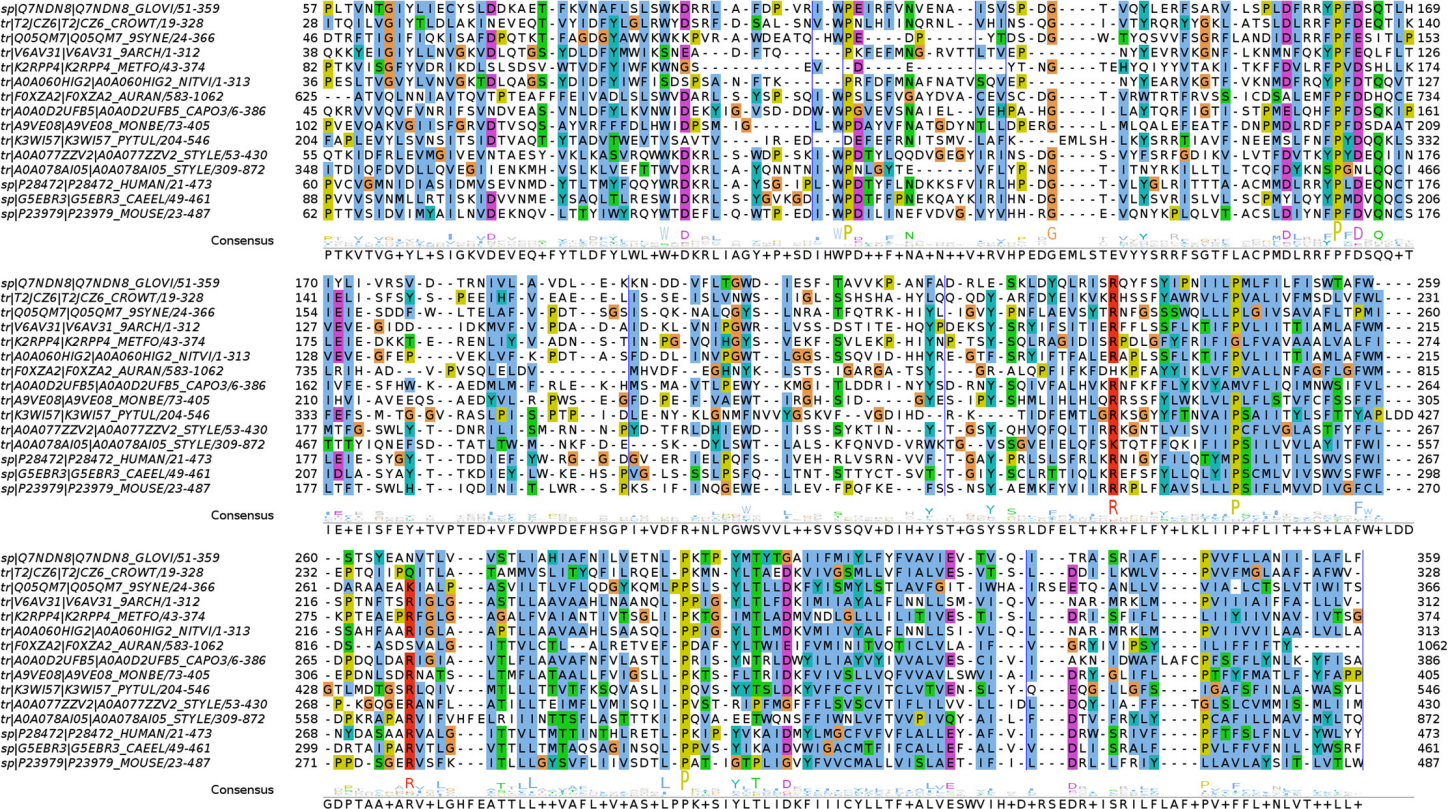
Subset of large multiple sequence alignment. Contains 11 novel pLGIC sequences from bacteria, archaea, and eukaryotes. GLIC, nematode GluCl, mouse serotonin, and human GABA_A_ *β*3 receptors are included for comparison. Residues are colored by type according to the ClustalX scheme. Unconserved regions are hidden and indicated by blue, vertical lines. Species names are abbreviated in the figure. Bacteria: *Gloeobacter violaceus, Crocosphaera watsonii, Synechococcus sp*., archaea: *Thaumarchaeota archaeon, Methanobacterium formicicum, Nitrososphaera viennensis*, eukaryotes: *Capsaspora owczarzarki, Monosiga brevicollis, Pythium ultimum, Stylonychia lemnae*.

A sub-alignment of 15 sequences is presented in Fig 2. Several regions of the ECD appear poorly conserved, including some hydrophilic regions of the ECD that, in known structures, contribute to the *β*-sandwich core. This highlights the contrast between a tightly conserved fold and divergent sequences. In contrast, transmembrane segments align readily, constrained by the reduced alphabet of hydrophobic amino-acid residues. Aligning the M4 segment poses a challenge, as it is surrounded in N-term by the variable or absent cytoplasmic domain, and sometimes in C-term by sequence regions that do not belong to the common pLGIC architecture. As a result, predicted M4 helices only appear aligned when using the local alignment-based, iterative algorithms of MAFFT (E-INS-i and L-INS-i). A sequence profile for the balanced dataset is provided as S1 Fig.

### Conserved motifs and notable sequence features

**Fig 3:**
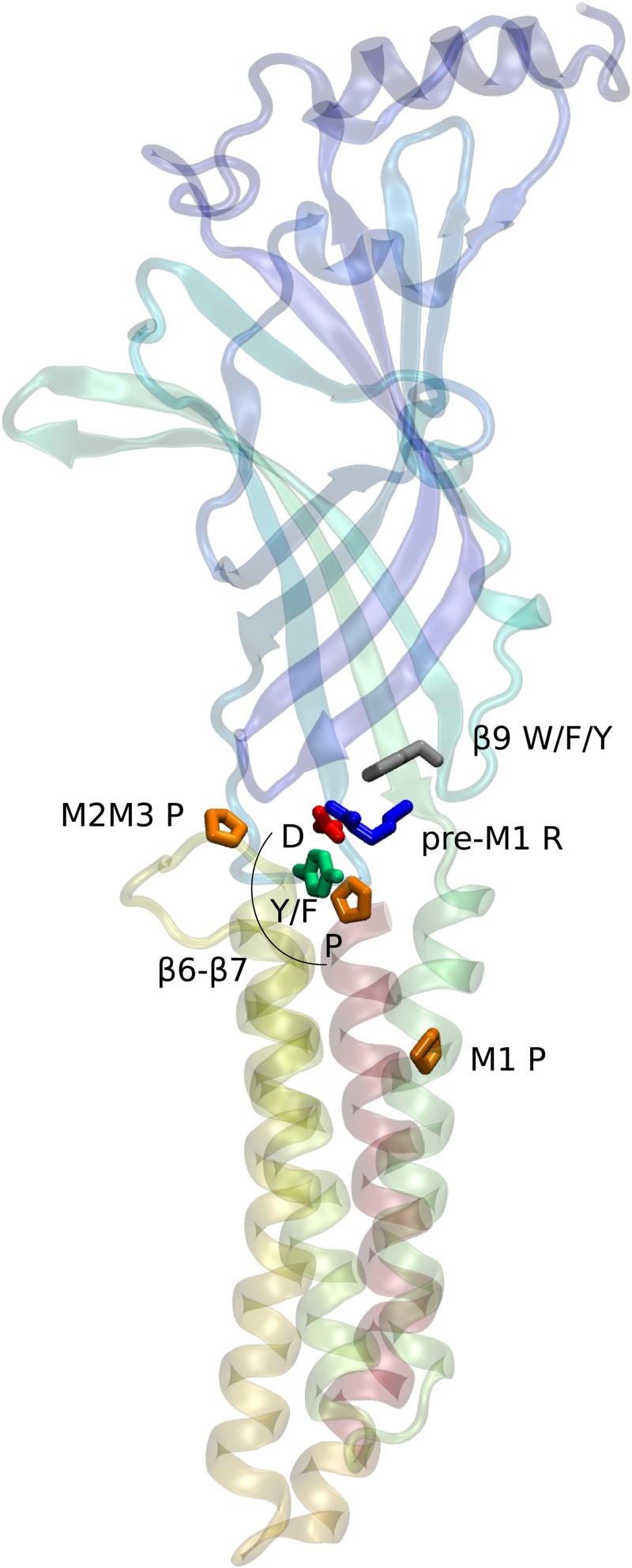
Location of the most conserved residues within the structure of a pLGIC subunit. One subunit of the homomeric GABA_A_ *β*3 receptor shown as cartoon, colored from blue to red along the sequence. Conserved residues listed in Table 1 are shown as sticks and colored by residue type (orange: Pro, grey: Phe, green: Tyr, red: Asp, blue: Arg).

The most conserved residues across the superfamily are listed in Table 1 and their three-dimensional arrangement is pictured in Fig 3. It is evident from the figure that these residues are clustered at the interface between ECD and TMD, within the coupling pathway between ligand binding and pore opening in known pLGICs. At the level of the superfamily, no conservation linked to more specific function emerges, reflecting the functional diversity of both ligand-binding and ion translocation. Furthermore, well-studied pLGICs including those of prokaryotes have demonstrated a high degeneracy of the sequence to fold to function relationship, with divergent sequences giving rise to a remarkably conserved fold, supporting a common functional pattern of ligand or pH-gated, anion or cation-selective transport.

**Table 1:** Summary of most conserved amino-acid residues throughout pLGICs in all taxons.

position	residue	frequency (%)	motif	substitutions	GLIC	GABA_A_ *β*3	5HT3
*β*6–*β*7	F	71^a^	F/YPxD^b^	Y 25%	119	143	142
*β*6–*β*7	P	100	F/YPxD	-	120	144	143
*β*6–*β*7	D	93	F/YPxD^c^	E 3%	122	146	145
*β*9	W	71^a^	ECD core^d^	F 16%, Y 11%	160	186	187
pre-M1	R	94	salt bridge^c^	-	192	216	218
M1 14’^e^	P	77^a^	M1 kink	G 5%	204	228	230
M2M3	P	88	M2M3 loop	G 3%	247	273	274

The table lists residues with conservation above 80% in our balanced set of 561 pLGIC sequences throughout the tree of life. Substitutions above 2% frequency with similar residues are indicated. To prevent bias due to fragmentary sequences, this conservation measure excludes gaps. All positions listed have a gap content below 2%. Residue numbering is given for three pLGICs crystallographic structures: *Gloeobacter* GLIC, human GABA_*A*_ *β*3 and mouse serotonin receptors.

^a^ Included because similar residues are more than 80% conserved.

^b^ Packed between aromatic M1 2’, *β*6–*β*7 proline, and bulky apolar M2 22’.

^c^ These two residues form a salt bridge.

^d^ This aromatic sidechain is buried in the hydrophobic core of the *β*-sandwich fold of the ECD.

^e^ See our proposed prime notation for M1 and M3 in the TM domain section below.

The highly conserved [F/Y]PxD motif forms the tip of the *β*6-*β*7 loop (“Cys loop”), and is the most recognizable signature of the pLGIC superfamily. In known structures, the aspartate residue forms a salt bridge with the equally conserved arginine of the pre-M1 linker. The proline residue is the single most conserved residue in our alignment (Table 1), and the only absolutely conserved residue throughout the superfamily.

The pair of cysteines flanking the Cys loop and forming a disulfide bridge are conserved in metazoans, and were noted by Tasneem et al. to be absent from bacterial sequences, where they are typically replaced with a polar residue for the first cysteine and with a hydrophobic residue for the second. We find that protist pLGICs fall under two separate categories: most contain a prokaryotic-like, Cys-less loop, but four species possess pLGICs featuring a *bona fide* Cys loop (annotations in Fig 1). A more precise relationship between those categories is elicited in the phylogenetic analysis described below.

We find Cys-less pLGIC sequences in invertebrate Metazoa from our representative set. To complete this surprising finding, we ran a taxonomically unrestricted search that yielded more Cys-less channels in metazoan species, including one cnidarian and one echinoderm, several nematodes, platyhelminths, annelids, and mollusks (bivalvia and gastropoda). Cys-less Pro-loop receptors are not found in any vertebrate species, and only in two chordates: the cephalochordate *Branchiostoma floridae* (amphioxus or lancelet) and the tunicate *Oikopleura dioica*. One Cys-less metazoan pLGIC was identified in the nematode *Dirofilaria immitis*[30] even before the discovery of prokaryotic pLGICs, yet that finding has not been widely publicized in the community. This channel (Uniprot ID Q70GM3) possesses a unique variant of the Pro loop sequence, with a YPFE motif (with E instead of the more common D) shifted by one residue towards the N-terminus. This trait is shared by four other nematode pLGICs, which form a molecular clade (see phylogeny below).

The ECD displays a number of partially conserved aromatic positions. Based on known structures, some belong to the hydrophobic core of *β*-sandwich structure, while others are expected to participate in the ligand-binding site where they might form cation-pi interactions. The latter are less conserved than the former, consistent with the fact that the ligand-bind motifs are more variable than the core architecture of the ECD, although the known set of binding site structures and corresponding ligands may only represent a fraction of the functional repertoire of pLGICs in unicellulars.

The most conserved position within TM helices is a proline in M1 that forms a kink in known structures. This kink has been shown to undergo a structural transition during the gating and desensitization processes in nicotinic receptors. [31]

Charged residues in pre-M2 position have long been documented to form a charge selectivity filter [32, 33]. In our dataset, the cationic residue at M2 0’ is found with 79% conservation (R and K combined). The previous two residues that form the classic metazoan motifs are more variable in prokaryotic sequences. M2 has one other relatively conserved position: Leu 9’ (75%), which can be substituted with a Phe (13%).

### Transmembrane domain: prime numbering for M1 and M3

Homologous residues in transmembrane helix M2 are conventionally compared across pLGICs using a standard prime numbering. The lack of a similar convention for M1 and M3 is impractical, as evidenced by published work on a glycine receptor [35] referring to an M1 residue as -26’, in M2 notation. Most prokaryotic pLGICs as well as some mammalian cases (5HT3) show indels within the M1M2 linker, making that notation non-constant across receptor families. The prime numbering used for M3 by Auerbach and coworkers [34] is defined for nAChR subunits only.

**Fig 4:**
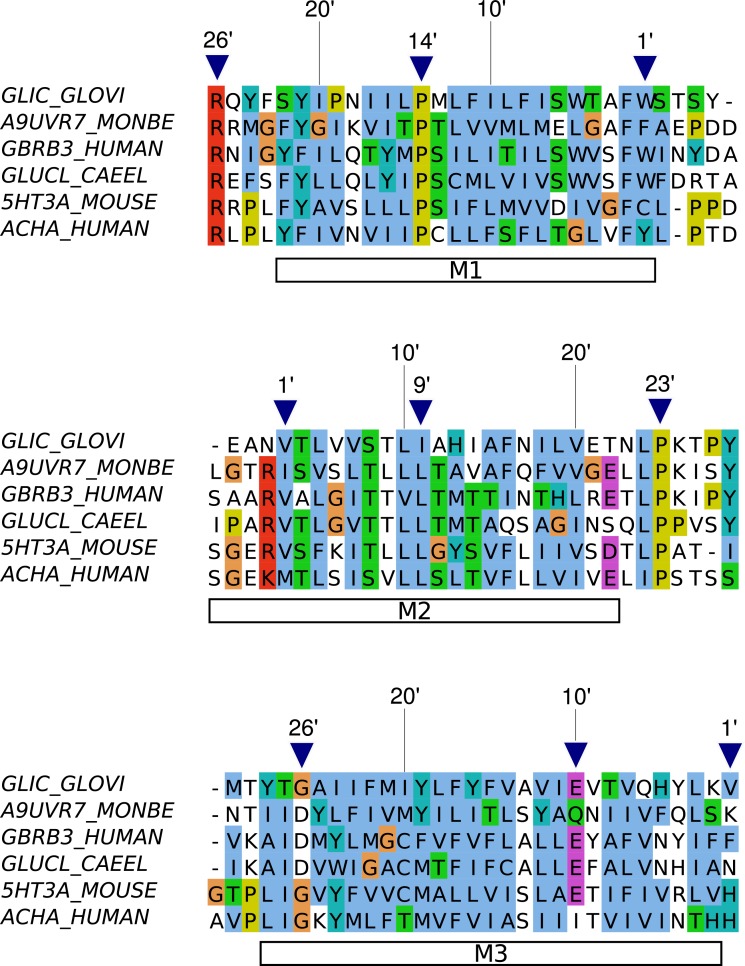
Prime numbering scheme for transmembrane helices M1 to M3. A sequence alignment for a set of pLGICs is shown annotated with a prime numbering convention in each helix, starting on the cytoplasmic side. Sequences are labeled with their abbreviated gene and species names, or Uniprot identifier in the case of the predicted pLGIC from the protozoan *Monosiga brevicollis*. The figure shows the existing convention for M2, generalizes that proposed for M3 in nAChR *α* subunits [34], and proposes a new convention for M1. Triangles indicate 1’ positions as well as conserved residues that may help anchoring other sequences.

Despite significant variability in transmembrane helices M1 and M3, we find that the alignments are robust enough to propose universal prime numberings for those two helices as well (Fig 4). We follow the convention of numbering residues from cytoplasmic to extracellular side.

In M1, we give the partly conserved C-term aromatic residue (GLIC W217) number 1’. Conserved residues include F 2’, P 14’, and extending beyond the helix, R 26’ (pre-M1 linker).

The M2M3 loop is well-known to be critical, and would benefit from a universal residue notation. Cationic channels typically display an insertion at the end of that segment, just before M3 0’. M2 numbering may be extended until position 25’ unambiguously, after which indels appear in some sequences.

For helix M3, we follow the convention started by Cadugan and Auerbach [34] for nicotinic receptor *α* subunits. As helix M3 is relatively divergent across families, we anchor the numbering by referring to motifs present in individual groups of pLGICs. The initial T/P/K position (GLIC T253, Torpedo nAChR*α* P272, GABA_*A*_ *β*3 K279) takes number 29’. Position 24’ is aromatic-rich (65% in balanced set, 89% in metazoan sequences only). Typical metazoan anionic channels have: K/T 29’, D 26’, C 20’, F 15’, E 10’. Typical metazoan cationic channels have P 29’, G 26’, Y/F 24’, polar 4’.

In eukaryotes, most acetylcholine receptors have a Cys at M1 10’, while most anionic channels have one at M3 10’. In GABA_*A*_ *α* and *γ* subunits, both are present, and predicted to be ideally positioned to form a potential disulfide link between M1 and M3. [36] The recent crystal structure of a GABA_*A*_ *β*3 homopentamer did confirm the structural predictions of that study, yet unfortunately did not provide a test of the hypothetical disulfide bridge, as *β*3 subunits lack the M1 cysteine.

The sequences for helix M4, although they broadly align together, show too little conservation for a global numbering to be reliable.

### Phylogeny of pLGICs

The maximum-likelihood prediction yields an unrooted tree. One approach to root it is to reconcile the molecular phylogeny and the phylogeny of species with the program Notung, minimizing a penalty reflecting the number of gene losses and duplications requested by each choice of root. The results from that analysis are ambiguous, as many edges receive the best root score. However, those likely roots lie within the same broad region of the tree, comprised of the deep connections between prokaryotic branches. Although the precise position of the root remains uncertain, this does not affect our conclusions below. The resulting unrooted tree is available online for download as well as flexible, searchable visualization at: http://itol.embl.de/shared/jhenin.

Eukaryotic pLGICs are predicted as monophyletic, although with low statistical support. That is mostly reflected in one clade of bacteria whose position in the tree is unstable, and which appears either close to or within the eukaryotic group depending on small variations in the data, in particular alignment filtering. We opted for a loose filtering scheme following the conclusions of Tan et al. suggesting that stricter alignments may reduce the accuracy of inferred phylogenies.[37] The same lack of statistical support was apparent in the tree published by Tasneem et al., only made more striking by a considerably smaller dataset wherein most relationships were *deep* relationships and therefore difficult to ascertain.

Eukaryotic proteins form two notable clades: Cys-less channels, including a group of Cys-less channels of invertebrate metazoans; and a Cys-loop clade. The Cys-less clade includes genes from protists and one group of Cys-less channels from metazoans, which is dominated by Lophotrochozoa but also includes a group of sequences from Platyhelminths, and a sister group of 5 sequences from Deuterostomia (Branchiostoma and Strongylocentrotus), together with an isolated sequence from Nematostella.

**Fig 5:**
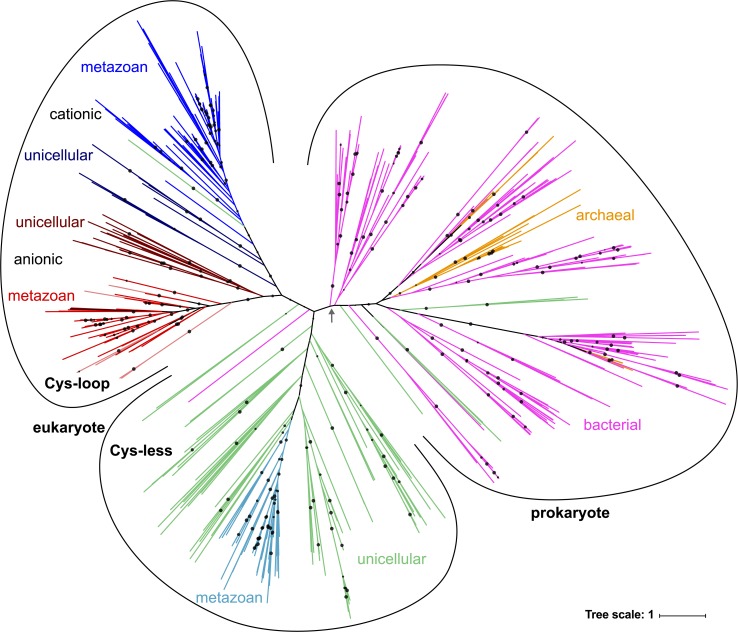
Inferred phylogenetic tree of the Pro-loop superfamily. Branch colors represent a combination of taxonomy and sequence features: magenta: eubacteria, orange: archaea, pale green: Cys-less pLGICs of protists cyan: Cys-less pLGICs of metazoans, blue: cationic-type Cys-loop of metazoans, dark blue: cationic-type Cys-loop of protists, red: anionic-type Cys-loop of metazoans, dark red: anionic-type Cys-loop of protists, pale red: anionic-type Cys-less of metazoans. Circles indicate SH support above 90%. The gray arrow indicates two bacterial branches of poorly-defined position, which sometimes group with eukaryotic sequences.

The Cys-loop group includes two subclades, one of which contains the anionic channels of metazoans, as well as protist Cys-loop receptors with a sequence signature for anion selectivity in the pore-lining M2 segment, the second contains cationic channels of metazoans and protist sequences with the equivalent signature for cation selectivity. Thus, the grouping of multicellular and unicellular genes within the Cys-loop clades is congruent with a very simple, 3-amino-acid sequence signature in the transmembrane domain (with the exception of one *Nemtostella* sequence, colored black in Fig 5, which has neither the typical cationic, not the typical anionic signature). Henceforth we will refer to those two groups as anionic-type and cationic-type Cys-loop receptors, respectively, although the ionic selectivity of their unicellular members is predicted from a simple sequence signature and phylogenetic grouping with bilaterian receptors, rather than characterized experimentally.

A few Cys-less sequences are also found within the anionic Cys-loop clade: we refer to them as “secondary” Cys-less pLGICs. They mostly belong to two groups of nematode genes, one of which includes the unusual Pro loop sequence of *Dirofilaria immitis*[30]. Isolated cases of secondary Cys-less sequences are two sequences in the spider *Stegodyphus mimosarum* and one in *Oikopleura dioica*. Note that a search for Cys-less sequences in all Metazoan genomes yielded no Cys-less pLGICs in other tunicate genomes, including that of *Ciona intestinalis*. A group of two Cys-less sequences from ciliate protozoa of the family Oxytrichidae (*Stylonychia lemnae* and *Oxytricha trifallax*) appear within the cationic Cys-loop clade.

Within prokaryotes, a clade of sequences belonging to Thaumarchaeota emerges, while other archaeal sequences are more interspersed with their bacterial relatives. Seven Cys-less sequences from protists appear within the prokaryotic branches (pale green in Fig 5), although always with SH support below 90%, indicating uncertain placement. Conversely, one isolated sequence from *Synechococcus* groups with eukaryotic sequences, and the bipartition separating it from other prokaryotes and Cys-les seukaryotes has a low SH support value of 68%.

## Discussion

### Pro-loop receptors

Confirming previous results [6, 17], we find that the proline residue forming the tip of the *β*6–*β*7 loop is the single most conserved position in the superfamily. This degree of conservation implies that it is subject to high evolutionary pressure: it is presumably essential in maintaining a conformation of the loop that is functionally critical. Based on a high-resolution structure of GLIC and a reinterpretation of crystallographic data on other pLGICs, this proline has been argued to be in a *cis* configuration in all known structures [12]. This suggests that the superfamily’s absolutely conserved, defining feature is not just a proline, but specifically a cis-proline residue, in line with a precise requirement on the local conformation of the loop. Rendon et al. [17] already noted this conservation and suggested renaming the Cys loop p-loop, however, the phrase has not gained adoption since, perhaps because of possible confusion with similarly named motifs. More closely mimicking the name “Cys loop”, however, we propose that “Pro loop” would be an accurate and unambiguous name for both the loop and for the superfamily.

The Pro loop as a whole is a highly conserved motif at the core of the conserved cluster within the ECD/TMD interface (Fig 3 and Table 1). This interface features two conserved prolines, two aromatic residues, and a pair forming a salt bridge between the Pro loop and the short *β*10–M1 linker. An explanation for the high selection pressure on these residues is that they maintain the subtle mechanical contact between the two domains in a way compatible with signal transduction across the interface. The fact that this conservation transcends receptors families with different functions is consistent with the persistence of signal transduction in chimeric receptors that mix those families [38–41].

The present results confirm that the cystine bridge closing the *β*6–*β*7 (“Cys”) loop is a secondary feature of pLGICs in some eukaryotes (including most metazoa and all vertebrates), rather than a primitive characteristic of the superfamily. The few reports of experiments investigating the role of these residues find them to be essential. Based on mutagenesis on the *α* subunit of *Torpedo* nAChR, Mishina et al. [42] hypothesized as early as 1985 that the pair of cysteine residues is “essential for maintaining the proper conformation of the extracellular region of the AChR molecule”, as it abolished binding of bungarotoxin to the surface of injected *Xenopus* oocytes. A mutagenesis study on glycine receptor subunit *α*1 indicated that mutation of one cysteine (198) to serine abolishes receptor expression to the cell surface, while mutation of the second cysteine (209) allows some expression to the cell surface, but still abolishes glycine-induced whole cell currents and strychnine binding [43]. In contrast, cysteine cross-linking experiments on glycine [44], GABA_*A*_[45, 46], and nicotinic [47] receptors found that a reducing agent had no significant functional effect on wild-type receptors, implying either that the Cys-loop disulfide is too stable to be reduced under the conditions of those studies, or that such a reduction has little impact on the fully folded receptors. In support of the second hypothesis, the fold of the ECD in GLIC and ELIC is essentially identical to that of Cys-loop receptors and stable in the absence of a disulfide. The hypothesis that the disulfide is necessary for native folding of Cys-loop receptors is neither validated nor contradicted by the existence of GLIC and ELIC, as bacteria have a different protein expression machinery; however, GLIC can be readily expressed in eukaryotic cells such as *Xenopus* oocytes [7]. The present data reinforces that notion, with the finding of native Cys-less pLGICs in eukaryotes (many protists and a few invertebrates). Although those are predicted proteins without biochemical or biophysical characterization, at least some of these sequences can be expected to yield functional proteins. If indeed the pair of cysteines is essential for folding Cys-loop receptors, then Cys-less pLGICs must have an alternate mechanism that forms and stabilizes the very same native fold. Thus, the appearance and subsequent conservation of the bridging cysteines remains partly unexplained. We postulate that functional Cys-less mutants of Cys-loop receptors could be designed based on Cys-less sequences, perhaps including additional hydrophobic residues stabilizing the core of the ECD.

### Congruency and incongruencies of molecular and species phylogenies

Some taxonomic clades are reflected in the molecular phylogeny, although often with exceptions. Those congruent groups include eukaryotes, and within those, metazoa, as three separate sub-clades of larger groups of eukaryotic pLGICs: cationic-type Cys-loop, anionic-type Cys-loop, and Cys-less.

Within unicellular eukaryotes, the distribution of different clades of pLGICs shows little correlation with cladistic groupings (Fig 1). We find no Cys-loop genes in Oomycetes, compatible with an early gene loss in that group. Other groups have too few representatives in our dataset to draw any conclusion.

Although Opisthokonta are the closest extant relatives of Metazoa, we find no Cys-loop gene in unicellular members of that taxon, but only Cys-less pLGICs; conversely, Cys-loop receptors are found in a wide range of eukaryotic taxons, pointing to either an early origin followed by frequent loss, or very widespread lateral transfers, or both. Some sequences of *Monosiga* do appear at the base of the Cys-less group of eukaryotic pLGICs, placing them close to the anionic group of Metazoan Cys-loop receptors. We note that the phylogenetic position of 2 out of 4 such sequences has poor statistical support.

The case of *Emiliania* has been pointed out as unusual: it is a “pan genome” [48] assembled from many strains, which explains the extraordinary number of pLGIC genes it contains (34 after excluding fragments), compared with any other genome outside Metazoa. Those sequences exhibit limited taxonomic congruency: although many of them form clades, others group with those of different protists. This may reflect either ancient divergence of those groups, or the uncertainty of the predicted deep relationships, highlighted by the large dataset from *Emiliania*.

One group of eukaryotic-like bacterial sequences including the GLIC and ELIC channels (gray arrow in Fig 5) shows an unstable position, appearing within the eukaryotic branch in some phylogenies inferred from intermediate data (not shown). Accordingly, its grouping in the tree presented here has poor statistical support.

Of note, the ten pLGIC sequences from Thaumarchaeota form a statistically robust clade, suggestive of a single gene acquired by an ancestor of that group. Taxonomic coverage of that group is, however, partial, indicating gene loss. These Thaumarcheota sequences are part of a clade of 16 sequences from Archaea. Eight other archaeal genes are split into several groups among bacterial relatives, possibly reflecting acquisition through several independent lateral gene transfers.

### Tentative evolutionary history

**Fig 6:**
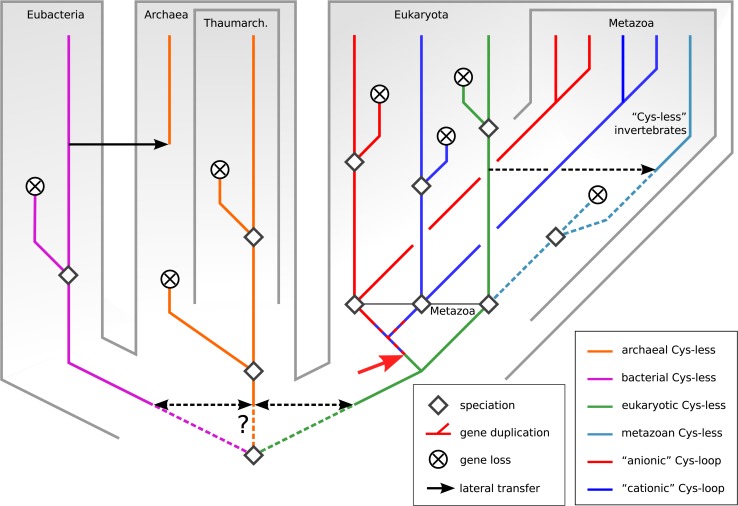
Schematic, putative molecular phylogeny of the Pro-loop superfamily. Each taxonomic category represents many species. Dashed lines indicate alternate hypotheses. Speciation events followed by short branches ending with gene loss depict the many unicellular lineages that have likely lost their Pro-loop receptors. In the case of metazoans, the cyan line indicates the clade of Cys-less Pro-loop receptors that survives in a few extant invertebrates. The red arrow indicates appearance of the Cys loop, presumably in an ancient unicellular eukaryote. Dashed red-blue lines describe the ancestral Cys-loop receptor, which may have been anionic or cationic-type.

Fig 6 presents a speculative, schematic evolutionary history of the pLGIC superfamily based on the molecular phylogeny of Fig 5 and including alternate hypotheses for some unresolved questions.

The scattered presence of pLGICs in Bacteria across several major phyla is intriguing. It points to either massive gene loss happening in most clades, or a number of lateral transfer events. It remains unclear in what taxon pLGICs originated, for three reasons: their sparse distribution in extant species, the uncertainty on deep phylogenetic relationships, and the resulting difficulty of precisely rooting the complete gene tree.

The origin of archaeal pLGICs is equally unclear, but again, requires a combination of lateral transfer and gene loss events given the sparse distribution and taxonomically incongruent phylogeny. The main novel information is the presence of pLGICs in 6 species of the relatively recent group Thaumarchaeota [25], whose pLGICs form a molecular clade.

The result that eukaryotic pLGICs are monophyletic is compatible with two simple hypotheses for their origin: either a single speciation event, namely the appearance of eukaryotes, or a single lateral transfer event to an ancestral eukaryote. Whichever of these two events occurred was followed by early duplication and differenciation into the extant pLGIC families, as well as frequent loss of some of those differentiated types of receptors (all of them, in some unicellular species). The high rate of loss in unicellulars might be explained by contraction of unicellular genomes [49]. The presence of both the cationic and anionic Cys-loop clades in unicellular eukaryotes pushes back the appearance of these clades to an early stage of eukaryotic evolution, before the appearance of metazoans.

This finding solves one question raised by Tasneem et al. a decade ago [6]: did eukaryotes acquire pLGICs “well before the emergence of the animal lineage”, followed by frequent loss in other eukaryotes, or was there “a single precursor for all the animal sequences”, from which “the massive radiation of the Cys-loop receptors occurred only after the animals branched off”? Those authors accurately added that this could only be answered after more eukaryotic sequences become available. Although the present data, is compatible with a single ancestor gene for all eukaryotic pLGICs, it indicates that some of the diversity of Cys-loop receptors—namely, the anionic and cationic branches—predates the appearance of metazoans.

The recruitment of pLGICs into synapses in Cnidaria and Bilateria was linked with an evolutionary pattern of successive gene loss and gene expansion events [50]. In those metazoans, a dependence on rapid synaptic transmission may have enshrined the corresponding pLGICs and prevented the pattern of drastic gene loss that prevails in unicellulars. Still, it appears that some Bilateria did lose individual families of pLGICs, such as GABA(A) receptors in the case of *Schistosoma mansonii*[51].

Among the protists identified, the Choanoflagellate *Monosiga brevicollis*, one of the closest known relatives of metazoans, prompts new hypotheses about the origin of metazoan pLGICs. One such hypothesis is that extant metazoan pLGICs have direct ancestors in protists of the clade Holozoa. Unfortunately, the phylogenetic positions of *Monosiga* pLGICs have poor statistical support, not allowing for clear conclusions. We should note that discussing the evolution of individual genes in protists is all the more difficult that the deep phylogeny of eukaryotes is subject of active research, and their taxonomy somewhat unstable [52].

We find that “true” Cys-loop receptors form a monophyletic group within pLGICs, consistent with a character acquired once and conserved since. There are few cases indicating secondary loss of the cysteines, and conversely they are not found outside the Cys-loop clade. We cannot say whether all such genes were inherited linearly from a very ancient common ancestor, or appeared more recently, evolved in a more limited lineage, and were acquired by distant eukaryotic species through lateral transfer. All three pLGIC groups (Cys-less, anionic-type and cationic-type) are present in *Emiliania* (Haptophyte), *Aureococcus* (Heterokont), Ciliates, and Opisthokonta, including metazoans (Fig 1). Based on the present data, it is not possible to place the origin of Cys-loop receptors with certainty any later in the history of eukaryotes than the common ancestor of all those groups, which may well be the common ancestor of all extant eukaryotes.

Cys-less Pro-loop receptors are unexpected in metazoans. Most of them belong to Lophotrochozoa and form a statistically well-supported clade together with Cys-less protist channels, and a few are “secondary” Cys-less pLGICs within the Cys-loop clade. Two possible histories can be proposed for the Cys-less eukaryotic clade: 1) They derive from an ancestral receptor that was lost in other metazoans, possibly because it did not undergo the same selection pressure as Cys-loop receptors, for example due to a function outside the nervous system. 2) These metazoans with Cys-less pLGICs acquired the Cys-less form through a lateral transfer event from a unicellular euaryote. The biology of such channels is not documented, as only one member is mentioned in the literature [30] with a report that an RNAi experiment yielded “no obvious phenotype”. The channel in question belongs to the nematode *Dirofilaria immitis* and is predicted to belong to the anionic Cys-loop clade, which implies that it probably results from a secondary loss of the cysteine residues. Based on that result, together with their evolutionary grouping with protist proteins, we predict that Cys-less invertebrate pLGICs could have a non-synaptic expression pattern.

Incongruencies between the present molecular phylogeny and that of species suggest several lateral transfers in unicellular organisms. This notion is compatible with the ecology of many of the pLGIC-containing unicellular species, many of which share biotopes, or are even symbionts or parasites of larger organisms. Microbial organisms with pLGICs include many marine species, among which some of the most abundant species of photosynthetic plankton, both prokaryotic (*Prochlorococcus* and *Pelagibacter*) and eukaryotic (*Emiliania*). *Teredinibacter turnerae* is an endosymbiont of mollusks [53]; *Chlorella* is an endosymbiont of freshwater unicellular organisms such as *Paramecium bursaria*. In general green algae of the *Trebouxiophyceae* class encompass endosymbionts of many organisms including mollusks. One species of Synechococcus was recently shown to accumulate in cells of the oyster *Crassostrea gigas* in a manner compatible with endosymbiosis [54]. Cases of photosymbiosis (symbiosis with a photosynthetic guest) exist between Platyhelminth and green algae [55]. Such symbiosis relationships with metazoans raise the possibility that Cys-less receptors may have been absent from the metazoan common ancestor, and were instead acquired through an ancient lateral transfer event.[56]

### Evolutionary biology of pLGICs in unicellular organisms

Among Cys-loop receptors, the presence of two clades including respectively the cationic and anionic members suggest a classification of protist Cys-loop receptors into a cationic-like and an anionic-like group. In contrast, the present phylogenetic study gives no indication on the potential ion selectivity of Pro-loop channels outside the Cys-loop clade, whether in eukaryotic or prokaryotic organisms. As the number of known pLGICs in unicellulars grows, it becomes more striking that we know no biological role for any of them. The *in vivo* function of GLIC, although by far the best-characterized prokaryotic pLGICs, is unknown. This is largely explained by the difficult to grow *Gloeobacter* in the laboratory. The ELIC channel is sensitive to GABA; it has been hypothesized that its function is linked to degradation of amino-acids in plant roots by *Dickeya dadantii*[8].

Chimeric receptors [38, 40, 41] and reverse-selectivity mutants [33, 57] have shown that the respective structures of the ECD and TMD can dictate ligand and ion selectivity independently, which implies that these two domains may, in principle, respond independently to evolutionary pressure regarding either ion or ligand specificity. The conserved core pictured in Fig 3 may then be seen as a universal adapter that allows these two modular structural elements to communicate after perhaps two billion years of independent evolution. In practice, the phylogeny of Cys-loop receptors reflects an early differentiation of anionic and cationic channels, followed by that of large clades of receptors with unique ligands in early Bilateria or earlier [58]. Ligand sensitivity shows more evolutionary mobility than ion selectivity: indeed, cases of convergent evolution of ligand sensitivity has been discussed in some Cys-loop receptors. Dent mentions nematode acetylcholine-gated chloride channels, whose neurotransmitter binding site “represents a unique structural solution to the problem of binding acetylcholine” [21]. Cases of homoplasy have also been reported for glutamate sensitivity of GluCl receptors [20, 59].

The M2 helix, like the ECD, is variable, but in metazoans it exhibits either of the GE[K/R] and A[R/K] motifs, which correlate with ionic selectivity (respectively cationic and anionic) [4]. For prokaryotic and protist sequences, this location in the alignment is in general not sufficient to predict ionic selectivity, as the residues of the two motifs are often substituted, only the final, basic residue being conserved.

### Limitations of this study

The high divergence of the superfamily makes both the search for homologues and subsequent sequence alignment challenging. Homologous sequences could exist in the currently sequenced genomes and yet evade detection for two reasons: genome assembly errors resulting in the transcript being incorrectly predicted or not at all; or a high degree of divergence making similarity to other pLGICs undetectable. The main argument pointing to the robustness of the search is the mostly similar sets discovered by Psi-BLAST and by HMM-based methods such as HMMer and InterPro.

While the Pro-loop motif Y/FPxD is found to be the single best marker of the superfamily, it could be argued that this reasoning is circular, as that motif contributes significantly to the statistical model built and used to detect pLGICs. It is hence entirely possible that pLGIC sequences divergent enough to be missing this motif would defeat remote homology searches and escape detection entirely, although a handful of sequences are detected although they lack the motif. One would expect to find a hint of this phenomenon in “gray area” sequences that are neither similar enough to known pLGICs, nor different engouh from them to conclusively decide whether they belong to the superfamily. In practice, the edge cases are mostly fragments, or appear to combine fragments of pLGIC sequence with unrelated fragments, and may reflect unreliable genome assembly rather than actual gene variants. One limitation of a full-length search including the transmembrane domain is that sequences with a similar membrane topology appear close to the detection threshold due to the lower sequence complexity of membrane-spanning segments. The requirement of a full-length match allows such cases to be eliminated.

The reliability of the inferred molecular phylogeny is limited by the evolutionary diversity of this set of sequences, and their ancient divergence. First, long divergence may lead to long-branch attraction. Second, mutation saturation may occur in the less conserved regions, leading to loss of pylogenetic signal. Finally, rooting the tree is made difficult by differences in evolutionary rates, which makes mid-point rooting meaningless, as well as a nonlinear evolutionary history. The most likely root lies deep within prokaryotic branches, yet root placement is approximate. Moreover, the relationship between the Pro-loop receptors of Archaea, Eubacteria, and Eukaryota is as unclear as the phylogenetic relationship between those clades themselves, that is, the placement of the root of the tree of life [60].

Due to the difficulty of aligning many divergent sequences, in this work we intentionally discarded terminal parts of the sequence before and after the two signature domains of Pro-loop receptors. Furthering the work started by Tasneem et al. [6] of studying the domain architectures of all members of the superfamily could lead to much insight into the roles of these proteins in unicellular organisms, and possibly non-bilaterian animals. Still, we note that the association of the classic pLGIC architecture with other domains has only been evidenced at the genome level so far: any biological conclusions would require experimental validation.

Our molecular phylogeny displays incongruencies with the species phylogeny, which carries some uncertainty itself. It could be argued that this reflects not just a non-linear evolutionary history but the dynamic nature of prokaryotic genomes, which challenges their very representation as a “tree of Life” [49].

### Perspectives for future work

These results leave many questions open, the deepest of which is the ancient evolutionary history of pLGICs. In what taxon did they appear? How were they acquired by ancient eukaryotes?

Co-evolution analysis may point to evolutionary networks: binding sites, interfaces between subunits or between TM helices, signaling network between the ECD and TM domains. At the genome level, it would be interesting to search for co-evolution of pLGIC genes with genes involved in the nervous system in animals.

Since the TMD ad ECD seem to dictate ion and ligand specificity, respectively, recombining these segments among paralogous genes may have been an evolutionary path to functionally novel receptors. This could be detected as incongruencies between phylogenies inferred separately from each domain, although isolated TMD sequences are likely to yield poor phylogenetic signal, making the analysis challenging.

The number of whole sequenced genomes for unicellular eukaryotes is still small. As more data becomes available, it should become possible to confirm or falsify the hypotheses put forth here.

## Methods

### Searching protein sequence databases for pLGIC homologues

In the spirit of Rendon et al. [17], we tested three independent approaches: iterative BLAST (Psi-BLAST [61]) and two methods based on hidden Markov models: HMMer [62], and Interpro [63]. Searches were run on the Uniprot SwissProt and TrEMBL databases, and NCBI Refseq and NR, using searches in larger and less well-curated databases to complete results from the smaller ones.

Psi-BLAST was found to be the most sensitive method by Rendon et al. We ran an iterative psi-BLAST [61] search (max. target sequences:5000, BLOSUM 45 matrix, e-value threshold: 1). Due to the over-representation of metazoan proteins in databases, unrestricted searches result in Position-Specific Scoring Matrices (PSSM) that are, in practice, characteristic of metazoan sequences. To avoid such a bias, the search was restricted by excluding the kingdom Metazoa.

We stopped at the third iteration due to a sharp increase in the number of false positives (detected as explained below). To mitigate the low specificity of the psi-BLAST search, we used two methods that were expected to be more specific: a domain-based search in InterPro [64] and an HMM-based search with HMMERsearch [65].

The HMM-based search was run using the HMMer server (hmmer.janelia.org) [62]. The initial input was the modest-sized alignment of metazoan and bacterial pLGIC sequences published by Tasneem et al. An HMM profile was created from this alignment and used to screen the Uniprot database.

As protein databases contain many thousands of metazoan pLGIC sequences, an unrestricted search yields a very large dataset that is heavily biased toward the Metazoa kingdom. To avoid that imbalance, we restricted the metazoan search space to a set of 31 representative metazoan species (S1 Table). The resulting dataset still contained 2400 metazoan sequences, and only about 300 sequences outside of Metazoa. A second HMM was constructed by randomly pruning the metazoan data down to 200 sequences, which formed a set of 500 sequences when joined with all microorganism sequences. This sub-sampled dataset is more balanced with respect to taxonomic distribution, which was expected to reduce biases in the alignment or sequence profiles due to the over-representation of Metazoa. The less focused HMM obtained by the alignment of this dataset was used as query in a new search with HMMsearch on Uniprot database with the Metazoa kingdom excluded.

A final search performed on the larger NR database provided hits that represented new species: only those hits were added to the Uniprot-derived database.

Separately, a domain-based search was performed using three InterPro signatures [63] common to the all pLGICs: the family signature IPR006201 (Neurotransmitter-gated ion-channel), and both individual domain signatures IPR006202 (Neurotransmitter-gated ion-channel ligand-binding domain) and IPR006029 (Neurotransmitter-gated ion-channel transmembrane domain). Hits from that search not retrieved by HMMer, typically because they belonged to subsets of Uniprot that were not available for scanning byHMMer, were retrieved, validated, and added to the dataset.

### Hit validation

A hit validation procedure was performed, combining reverse BLAST, a topological prediction, and pairwise alignment with the sequences of GLIC and GluCl. A hit to be validated as a true positive if it fulfilled at least two of the following criteria:

- A reverse BLAST starting from that sequence returns known pLGIC sequences in the first hits (keeping the same parameters as in the initial psi-BLAST search).
- The membrane as predicted by TMHMM [66] is compatible with a pLGIC subunit, or at least a topology compatible with a truncated pLGIC sequence. To account for the uncertainty of the topology prediction, and based on tests on known true positives, we used a threshold score of 0.2 to consider a residue as potentially part of a transmembrane helix, taking into account the fact that pore-facing helix M2 is less hydrophobic and thus not consistently detected as transmembrane.
- the sequence aligns well with GLIC, GluCl and other known pLGICs.

Our final dataset contains all sequences from microorganisms sequences and a representative group of 31 metazoan species. This still yields a large number of metazoan sequences (2088), mainly because animal genomes typically contain many pLGIC paralogues. For some applications, metazoan sequences were sub-sampled as described above to form a more balanced set of 500 sequences when joined with all microorganism sequences. This sub-sampled dataset is more balanced with respect to taxonomic distribution, which was expected to reduce biases in the alignment or sequence profiles. This set was complemented with all Cys-less metazoan sequences that were detected by a regular expression test on all hits, finding regions that matched the Pro-loop motif, but lacked one or both of the cysteines.

### Multiple sequence alignments

Due to the low quality of some sequences from uncurated databases, including sequences of very different lengths, some manual filtering was needed to enhance the quality of the alignment. Sequences that contained only small homologous fragments or large foreign insertions within conserved regions were removed. Extraneous domains at the N and C termini were truncated, either before a segment that aligned with the ECD of known pLGICs, or after the predicted fourth TM helix. This yielded sequences of more comparable lengths, and improved alignments.

Several alignment methods were assessed on our datasets: M-Coffee [67], MAFFT L-INS-i and E-INS-i [68], Muscle [69], HMMalign [66], and Promals3D [70]. The final alignment was produced with MAFFT version 7.266 [68], using the E-INS-i algorithm, which makes minimal assumptions about the sequence set, with up to 1000 steps of iterative refinement, and the BLOSUM62 matrix, which has been shown to exhibit better mathematical consistency than BLOSUM30 (no violations of the triangle inequality) [71]. Alignments were visualized using Jalview [72], annotating the alignment with the topological prediction from TMHMM [66] to validate the alignment of transmembrane segments.

Alignments was validated by combining different assessment criteria:

- comparison with pairwise alignments of well-known pLGICs, including structural alignments;
- lack of indels in key secondary structural elements, notably transmembrane helices;
- proper alignment of transmembrane helices as predicted by TMHMM; helix M4 proved particularly sensitive to alignment parameters.

### Phylogenetic inference

The alignment to be used for phylogenetic inference was filtered using TRIMAL [73] using the *gappyout* setting.

When developing the analysis workflow and working with intermediate datasets, we used FastTree 2 [74] to infer near-maximum-likelihood phylogenies extremely rapidly (less than half a minute for our data, on a single core of a desktop workstation).

After experimenting with filtering by TRIMAL [73] and GBlocks [75], and aware that strict filtering may worsen the accuracy of phylogenetic inference [37], we opted for a loose (default) filtering by Noisy [76], which aims at removing homoplasic positions that would drive phylogenetic inference into error. As the resulting alignment was still highly gapped and contained positions that were not reliably aligned, we filtered out sites with more than 50% gaps.

Assessment of the dataset by ProtTest [77] found the LG amino-acid substitution model [78] with gamma-distributed rates and the observed amino-acid frequencies (LG+G+F) to be optimal for this dataset. The maximum likelihood tree was inferred by RAxML, using LG+G+F, using subtree pruning and regrafting moves (SPR). Branch support was estimated by the Shimodaira x2013;Hasegawa approximate likelihood ratio test (SH-aLRT)[79] as implemented in RAxML, because ML bootstrap is known to be overly conservative [79].

Notung 2.6 [80] was used to reconcile the gene tree with the phylogenetic tree of pLGIC-possessing species, extracted from the tree of life of NCBI Taxonomy. The approach aims at placing the root of the gene tree to minimize the number of gene duplications and losses in the overall evolutionary model.

Phylogenetic trees of pLGIC genes were visualized and rendered in interactive Tree of Life [81]. Species trees were rendered with Archaeopteryx [82].

## Supporting Information

S1 Dataset Multiple alignment of pLGIC sequences from metazoans, archae, bacteria, and unicellular eukaryotes. (ZIP)

S1 Fig Sequence profile logo for the alignment of the balanced set of pLGIC sequences including prokaryotes and eukaryotes. (PDF)

S1 Table List of the 31 representative metazoan species with their taxid and Uniprot species identifier. (PDF)
