# Supplementary material for: Evolution of Pentameric Ligand-Gated Ion Channels: Pro-Loop Receptors
Source: PLoS One. 2016 Mar 17;11(3):e0151934. doi: 10.1371/journal.pone.0151934 (PMC4795631; doi:10.1371/journal.pone.0151934)
Supplement: S1 Fig — (PDF) [file pone.0151934.s002.pdf]

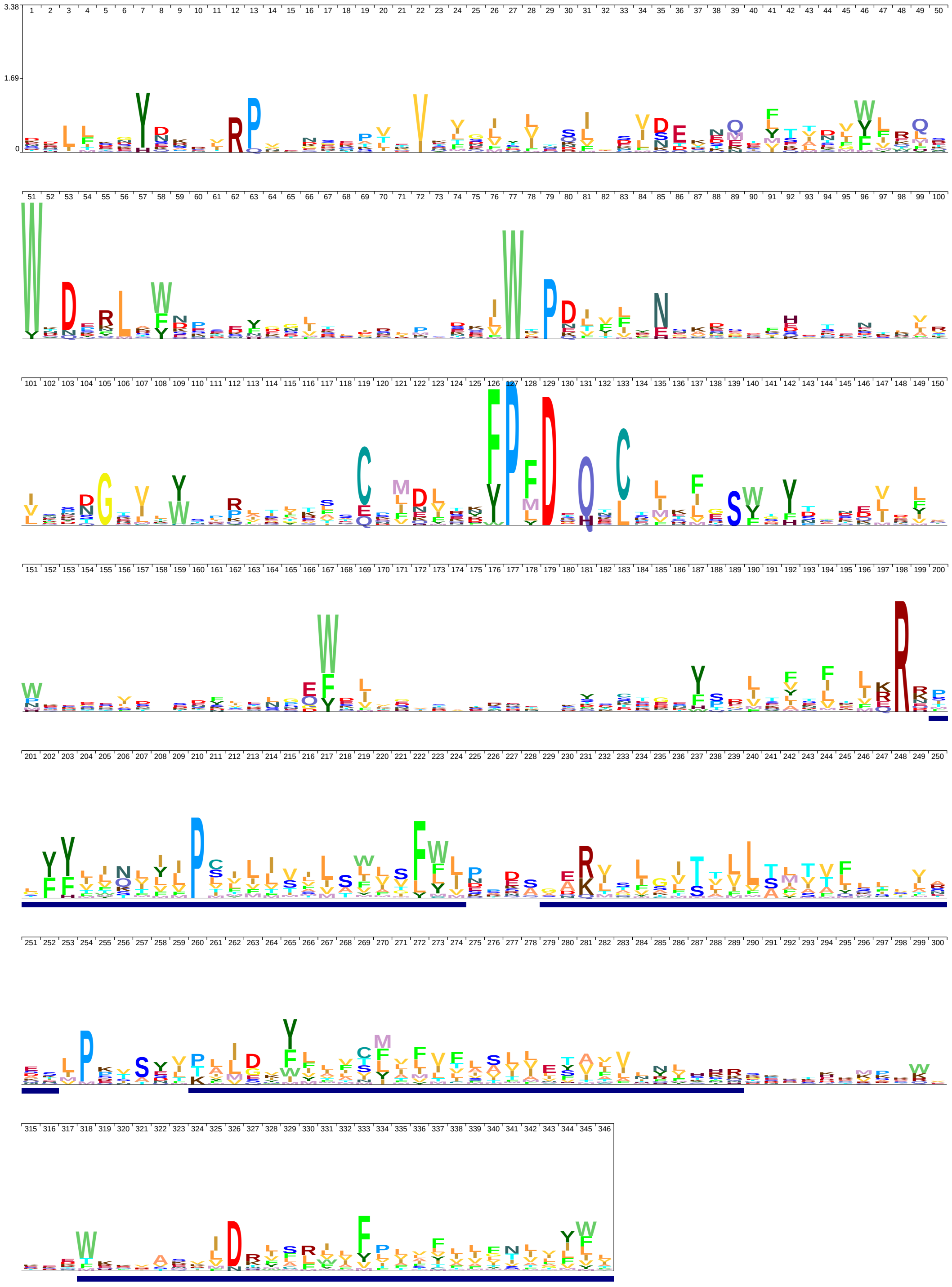

Figure 1: Sequence profile logo for the alignment of the balanced set of pLGIC sequences including prokaryotes and eukaryotes.
