# Supplementary material for: Evolution of Pentameric Ligand-Gated Ion Channels: Pro-Loop Receptors
Source: PLoS One. 2016 Mar 17;11(3):e0151934. doi: 10.1371/journal.pone.0151934 (PMC4795631; doi:10.1371/journal.pone.0151934)
Supplement: S1 Table — (PDF) [file pone.0151934.s003.pdf]

Species Name	taxid	Name abbreviation
Anas platyrhynchos (duck)	8839	ANAPL
Anolis carolinensis (anole lizard)	28377	ANOCA
Apis mellifera (honeybee)	7460	APIME
Aplysia californica (mollusc)	6500	APLCA
Brugia malayi	6279	BRUMA
Caenorhabditis elegans (nematode)	6239	CAEEL
Canis lupus (dog)	9615	CANFA
Capitella teleta (annelida)	283909	CAPTE
Chrysemys picta (turtle) (no sequence retrieved)	8479	CHRPI
Ciona intestinalis (ciona)	7719	CIOIN
Crassostrea gigas (mollusc)	29159	CRAGI
Danio rerio (zebrafish)	7955	DANRE
Drosophila melanogaster (droso)	7227	DROME
Echinococcus granulosus (platyhelminthes)	6210	ECHGR
Gallus gallus (chicken)	9031	CHICK
Helobdella robusta (annelida)	6412	HELRO
Homo sapiens (man)	9606	HUMAN
Hydra vulgaris (hydra)	6087	HYDVU
Latimeria chalumnae (coelacanth)	7897	LATCH
Monodelphis domestica (opossum)	13616	MONDO
Nematostella vectensis (medusa)	45351	NEMVE
Ornithorhynchus anatinus (platypus)	9258	ORNAN
Pelodiscus sinensis (turtle) (no sequence retrieved)	34908	PELSI
Petromyzon marinus (lamprey)	7757	PETMA
Saccoglossus kowalevskii (hemichordata)	10224	SACKO
Sarcophilus harrisii (Tasmanian devil)	9305	SARHA
Schistosoma mansoni (platyhelminthes)	6183	SCHMA
Strongylocentrotus purpuratus (ursine)	7668	STRPU
Takifugu rubripes (fugu)	31033	TAKRU
Trichoplax adhaerens (trochoplax) (no sequence retrieved)	10228	TRIAD
Xenopus tropicalis	8364	XENTR

Table 1: List of the 31 representative metazoan species selected with the taxid and the Uniprot species identifier.
